# An Assessment of the Strength and Physical Properties of Edible Tableware from Flax Seed and Flaxseed Cake

**DOI:** 10.3390/ma17225510

**Published:** 2024-11-12

**Authors:** Dariusz Andrejko, Agata Blicharz-Kania

**Affiliations:** Department of Biological Bases of Food and Feed Technologies, University of Life Sciences in Lublin, Głeboka 28, 20-612 Lublin, Poland

**Keywords:** edible tableware, plates, bowls, by-products, flaxseed cake, mechanical properties

## Abstract

Alternatives to traditional disposable plastic tableware are constantly sought. The aim of the study was to assess the possibility of using oilseeds and their press cakes for the production of edible tableware. Edible vegan plates (P) and bowls (B) were produced. The basic ingredients used for production were flax seeds (S) or flax press cake (C). Plates made using press cakes under a pressure of 3 kg deformed to a lesser extent than those containing seeds. However, they were more susceptible to crumbling during shaking. The colour of the tableware made on the basis of flax press cakes was lighter and was characterised by a higher chromaticity in the yellow and red direction. Significantly higher water absorption was characteristic of the tableware in which flax press cakes were used instead of flax seeds. The lowest water absorption (17.14%) after 30 min of soaking was recorded for the PS sample. After the test simulating the use of the tableware, a significant reduction in strength was observed overall (except for the PS test). The panelists rated the consistency and palatability of the PS, BS and PC tests very similarly. The highest overall acceptability was noted for the BS and PC tests. In conclusion, the development of edible bowls and plates made from flax seeds or flaxseed cake is an alternative solution for the production of environmentally friendly tableware.

## 1. Introduction

Recently, there has been an increase in demand for convenient food, which can be consumed almost anywhere [1,2]. The production and sale of such products requires the use of a large amount of disposable tableware and cutlery [3]. So far, these have mainly been plastic tableware. In general, the polymers usually used to produce disposable tableware are recyclable. However, in practice, only some of the materials are recycled [4,5]. The safety studies carried out have shown that plastics used to produce tableware and food packaging should be neutral materials that do not pose a threat to the consumer’s health [6]. However, the global problem of increasing plastic production causes significant environmental pollution. Observed climate changes and the desire to meet the assumptions of a circular economy have forced the introduction of a recycling system in the area of food packaging waste. There has also been an observed demand from consumers for environmentally friendly packaging materials made of biocomposites with biodegradable properties [7].

One of the directions of research has become the development of not only biodegradable tableware or packaging, but also in accordance with the principle of “zero waste” edible tableware [8,9,10,11]. This type of innovative solution will help to significantly reduce the amount of waste generated. Most of the recipes for edible tableware developed so far are based on the use of cereals flour or bran [11,12,13,14]. An innovative solution, perfectly in line with the assumptions of sustainable food production, would be to produce edible packaging based on by-products. The available research on this subject is quite sparse; several attempts have been made to use plant residues for the production of edible tableware, but by-products were treated as an additional ingredient. In the study by Choeybundit et al. [15], cutlery was produced with isolated soy protein and the addition of 5 to 20% crude morning glory stem fibre. The authors prepared a biocomposite by mixing the by-product with protein isolate and glycerol. The improvement of the mechanical properties and the reduction of water absorption were confirmed in samples with a higher addition of crude morning glory stem fibre. Matheswari and Arivuchdar [9] produced cups from a mixture of flours with the addition of peanut cake, apple pomace, beetroot and molasses—up to 20% in total. A total of one hundred grams of dough were formed, cast on top of a steel cup and baked at 166 °C for 15 min. Yet another by-product, brewer’s grain, was used to produce bowls [16]. The content of the additive was less than 10%. The hardness of the edible bowl (S4) was significantly increased (almost three times). Moreover, as observed in the preliminary soil degradation test, the developed bowl was biodegradable.

Siddiqui et al. [17] were the only ones to produce an edible spoon based on by-products (100% mosambi peel). The cutlery obtained met consumer expectations but was characterised by very high water absorption. No other experiments have been conducted to date describing the possibility of producing edible tableware consisting mainly of plant residues. One of the appropriate by-products could be oilseed cakes. In addition to the ecological aspect of using by-products, the impact of these raw materials on the nutritional and sensory properties of edible tableware is also important. Fruit pomace is a valuable source of fibre and polyphenols and is mostly characterised by a sweet taste and pleasant aroma. In turn, oil seed cake contains a large amount of protein, which can significantly improve the nutritional value of edible tableware. On the other hand, the use of food industry by-products for the production of edible tableware or packaging will probably result in a change in their functional properties, such as strength or water absorption.

The aim of the study was to assess the possibility of using oilseeds and their cake for the production of edible tableware.

## 2. Materials and Methods

### 2.1. Research Material

Flax seeds/flax cake, water, sunflower oil, salt, carob, cinnamon, pepper and wheat flour were used to produce the tableware (plates and bowls). The first stage was to obtain by-products of oil production by cold pressing flax seeds using a screw press (Sana, EUJ-702, Warsaw, Poland). The cake was ground in a laboratory knife mill (Chemland FW100, Stargard Szczeciński, Poland).

### 2.2. Methods of Preparation and Baking of Dough

The products were made according to the recipes in Table 1. The dough was prepared in a spiral mixer (KENWOOD food processor, Tokyo, Japan). Oil, flax mucilage and water were added to the dry products (previously ground). The dough was kneaded for 5 min. The prepared dough was appropriately shaped and baked (at 150 °C, for 12 min). The cake tin was then removed from the oven and left to cool to room temperature. The products obtained were of the following average dimensions [mm]: height: PS—14.7; BS—20.1; PC—15.1; BC—20,4; diameter: PS—70.6; BS—59.5; PC—71.0; BC—58.2; thickness: PS—5.2; BS—5.1; PC—5.2; BC—5,0. The plates and bowls were stored in airtight packaging at an ambient temperature (25 °C). The research material prepared in this way was subjected to further analysis.

### 2.3. Weight Load Test

The bowls and plates were subjected to a load of 3 kg. This test assesses the load-bearing capacity of the tableware, i.e., the ability to support a load of liquid or food during use. The test was conducted using a testing machine (Zwick/Roel, Z0.5, Ulm, Germany) with testXpert II v3.5 software. A flat attachment with a diameter of 100 mm was used. The loading was carried out at a speed of 2 mm·min^−1^. After the test was completed, the change in the height of the tableware was determined and the deformation coefficient was calculated, i.e., the change in the height of the tableware caused by compression [18]. The determination was performed three times.

### 2.4. Crumbling Losses During Shocks

The durability of a material is a measure of its resistance to shocks resulting from transport, various processes related to distribution and use. In order to determine the crushing losses during shocks, a shaking test was performed in which the tableware collided with each other and with the walls of the container. The measurements were taken using a laboratory shaker (Elmi W0301/7/00524, Riga, Latvia) at a rotational speed of 250 rpm, and the test time was 5 min. The amount of crumbling losses (CL) was determined based on a test performed in three repetitions.

Crumbling losses (CL) were determined from the following formula:

$$CL=\frac{m_{2}}{m_{1}}\times 100\;[\%]$$

where *CL*—crumbling losses [%], *m*_1_—mass of tableware before shaking [g] and *m*_2_—mass of crumbs after shaking [g]. Each sample was analysed in three repetitions.

### 2.5. Colour Parameters

The colour was determined using a 3Color^®^ SF80 (Marcq-en-Barœul, France) spectrophotometer. Each sample was analysed in five repetitions. The colour parameters were determined according to the CIELab system where L*—lightness, a*—colour chromaticity from green to red and b*—colour chromaticity from blue to yellow. The measurement was performed using the following light source: D65, observer: 10°, measuring head opening: 8 mm.

### 2.6. Strength Tests

The strength properties of the resulting tableware were tested after processing to simulate their use by the end consumer (the bowls were filled with warm water, while a mass containing 45 g butter and 5 g sugar was placed on the plates to replace a piece of cake). Strength tests were also carried out on “unused” tableware as a control sample. The mechanical properties of the prepared tableware were tested using a Zwick Roell Z 0.5 (Ulm, Germany) testing machine. A puncture test was performed using a flat cylindrical penetrator (5 mm in diameter). A head with a maximum pressure of 500 N was used. The test speed was 1 mm·min^−1^. The result was the maximum force recorded during the test. In this way, the resistance of the tableware to destruction was determined. The tests were performed four times.

### 2.7. Water Absorption Test

The samples were also subjected to a water absorption (WA) test. The test was conducted at an ambient temperature. The test measured the amount of water absorbed by the tableware over a 30-min period. The tableware were weighed, then placed in water in their entirety, removed after 5 more minutes, dried with tissue paper and their mass was determined [19]. Based on the obtained data, the WA was calculated as follows:

$$WA=\frac{W_{2}-W_{1}}{W_{1}}\times 100\;[\%]$$

where *W*_1_—weight before absorption and *W*_2_—weight after absorption. The determination was performed three times.

### 2.8. Sensory Analysis

The prepared tableware were assessed by a panel of ten team members 24 h after baking. Selected consumers were trained in the descriptive aspects of the test analysis. The panelists assessed the colour, aroma, palatability, consistency of the bowls and plates and overall acceptability. The results were presented on a 5-point structural scale (1—“I really don’t like it” and 5—“I really like it”). The panelists were employees (21–52 years old) of the University of Life Sciences in Lublin. The following criteria were taken into account when selecting the team members: good health, non-smoking and willing to participate. The study was conducted in a laboratory under LED lighting, at ambient temperature. The panelists were given mineral water as a neutralizing agent. The order of serving was random. The sensory characteristics of the tableware received were tested after processing consisting in simulating their use by the target consumer (the bowls were filled with warm water, while the plates were filled with a mass containing fat and sugar replacing a piece of cake).

### 2.9. Statistical Analysis

The obtained results were analysed using Statistica 13 software. The numerical values were subjected to one-way analysis of variance (ANOVA). The significance of differences was verified by Tukey’s test, at a significance level of α = 0.05. The results are presented as means ± standard deviation. The results of the sensory evaluation are presented in graphical form.

## 3. Results

**Figure 1:**
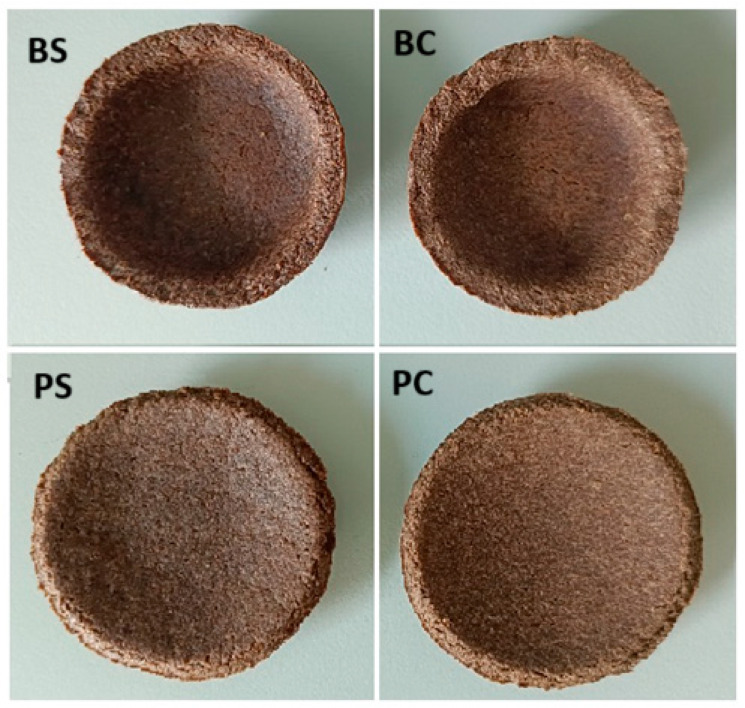
Appearance of the obtained edible tableware. PS—flaxseed plates, BS—flaxseed bowl, PC—flaxseed cake plates and BC—flaxseed cake bowl.

Figure 1 presents the appearance of the obtained edible tableware. The colour of the containers made of flaxseed cake seems to be lighter. The obtained tableware were tight (they did not let water through during the test simulating their use) and were not damaged after a load of 3 kg. All samples also retained their shape during the water absorption test—soaking.

The physical properties of the obtained tableware are presented in Table 2. The highest values of the deformation coefficient were noted for the PS sample. It should be noted that the changes in height were generally greater for the tableware in the shape of plates. The deformation of the bowls loaded with the same mass was more than twice lower. The degree of deformation of the tableware was also influenced by the ingredient used for making the dough. The material made with the use of cake deformed to a lesser extent than the one containing the addition of seeds; however, for the bowl-shaped tableware these differences were not statistically significant.

The crumbling losses were small, up to 0.12%. Higher CL values were noted for tableware made on the basis of flax cake. Statistical analysis confirmed a significant effect of the ingredient used on the amount of crumbling losses only for plates. Tests of tableware with the same composition did not show an effect of the shape of the container on the value of the CL parameter.

The brightness of biomaterials made from flaxseed cake was higher compared to the sample containing flaxseed. The a* and b* colour parameters were significantly different depending on the type of material tested. The highest values of redness and yellowness were observed for the BC sample. The b* parameter values were significantly lower for products made from flaxseed. Analogous relationships were observed for the b* parameter, but the differences between the PC and BS samples were not significant.

**Figure 2:**
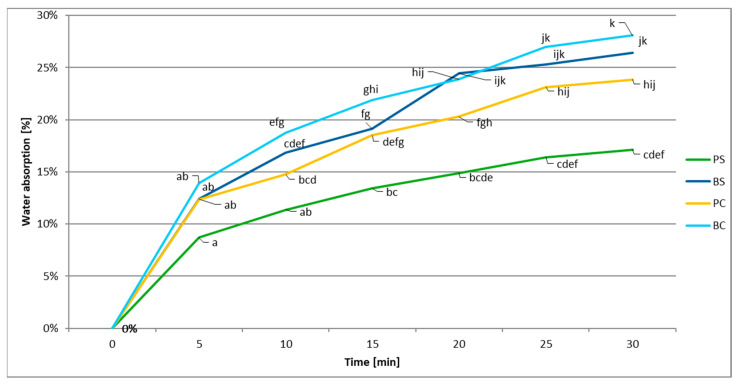
Water absorption of edible tableware. PS—flaxseed plates, BS—flaxseed bowl, PC—flaxseed cake plates and BC—flaxseed cake bowl. Different letters indicate significantly different values at a given point (Tukey test. *p* ≤ 0.05).

Figure 2 shows the changes in water absorption by linen tableware. The significantly higher WA results were noted for the bowls compared to the plates. Additionally, it should be noted that regardless of the shape of the tableware, significantly higher water absorption was characteristic of those containers in which flax seeds cake were used instead of flax seeds. The greatest changes in water absorption were noted in the range from 0 to 10 min. During the test, the lowest WA values were observed for the PS sample.

Before the test simulating the use of tableware, the PC biomaterial was characterised by the highest strength (Table 3). The strength of the remaining tableware before use did not differ significantly. After use, a significant decrease in strength was generally observed. The exception is the PS test, for which the changes were not statistically significant. The lowest strength was noted after use for a cup-shaped bowl.

**Figure 3:**
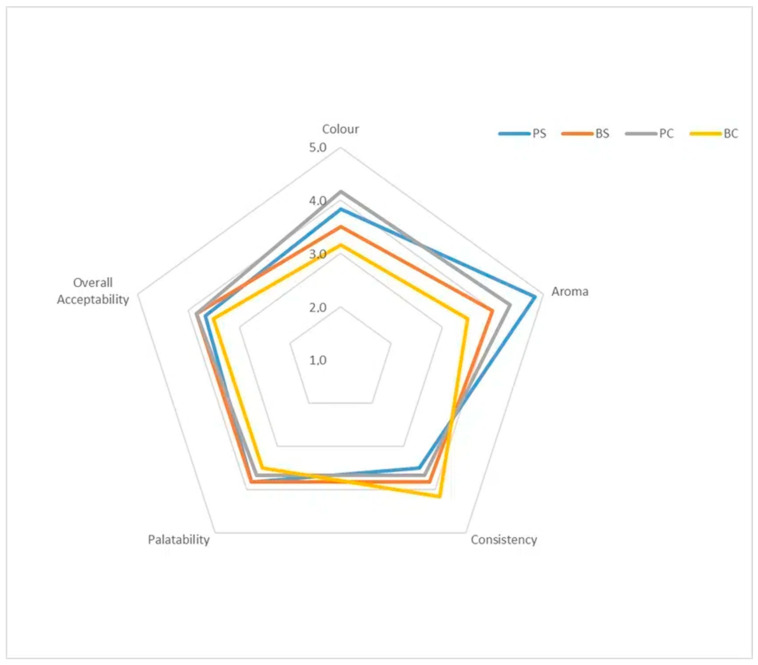
Sensory evaluation of edible tableware. PS—flaxseed plates, BS—flaxseed bowl, PC—flaxseed cake plates and BC—flaxseed cake bowl.

The attractiveness of the colour depended on both the form and composition of the tableware (Figure 3). The PS sample was rated the best, while the BC sample was rated the worst. The bowls made of flaxseed cake were also considered the least attractive in terms of aroma. According to consumers, the most pleasant aroma was felt when eating plates made of flaxseed. The panelists gave very similar ratings to the consistency, palatability and overall impressions of the PS, BS and PC samples. On the other hand, the consistency of the BC tableware was rated the best, which may be related to the lowest strength of this material, compared to the other samples. However, the BE tableware were considered to be the least palatable and generally the least acceptable.

## 4. Discussion

In the experiment, it was observed that biomaterials containing flaxseed cake bent less under a pressure of 3 kg than those made from flax seeds. Therefore, the use of by-products improved the mechanical properties of the tableware. In turn, in another study, spoons made on the basis of a concentrate of proteins and glycerol [15] bent to a greater extent after the introduction of plant residues—powdered morning glory stems. Such properties were also determined in the experiment by Buxxo and Jeetah [18], where by-products (pineapple and orange peels) were used to produce ecological cups based on hemp. It was observed that the introduction of plant residues to the biocomposite generally results in greater deformation of the obtained tableware.

However, it can be assumed that tableware with the addition of flaxseed cake were deformed under pressure to a lesser extent than those made from flaxseed, because flaxseed cake has a different structure and composition than flaxseed. First of all, flaxseed cake has much less fat than seeds alone. The high fat content makes the tableware more flexible and susceptible to deformation. Flaxseed cake is also rich in dietary fibres, which have structure-forming properties. These fibres, especially cellulose and lignin, increase the rigidity of the tableware structure, which makes them more resistant to pressure.

Greater crumbling losses for materials made on the basis of flaxseed cake also may be related to their chemical composition. The Zoulias et al. [20] study confirmed that baked cereal products with lower fat content were harder and more crumbly than traditional products.

Material obtained from flaxseed cake was characterised by greater brightness compared to PS and BS samples. This may be due to the lighter colour of defatted oilseed residues. Such changes were observed, among others, in the studies of Malaviya and Yadav [21] and Rani and Badwaik [22].

Lower water absorption means that the tableware can be used for longer before it falls apart or changes shape and becomes ineffective. It was observed that the cups absorbed more water than the plates, which is probably due to their larger surface area. An increase in WA was also noted for tableware made from flaxseed cake. The lower WA for tableware made of whole seeds, compared to vessels made of flaxseed cake, may be related to the lower water adhesion on the surface of the samples due to the higher fat content. In addition, the seed residues contain significantly higher amounts of protein and fibre [23,24]. The chemical composition of the raw materials used to make the biomaterials increases water absorption. Previous studies confirm that increasing the fibre content resulted in a higher rate of water absorption [25,26]. Hence, oil seed cakes have a higher water absorption than whole seeds [21,27]. The results and relationships obtained in this study are similar to those presented by other authors [19,28]. In the study by Habla et al. [19], control cups made of 100% cassava absorbed less water than tableware containing an additional 30% of banana. After 30 min of soaking at ambient temperature, the WA for tableware containing the banana addition was 41.18%. This value was higher compared to the tableware obtained in the discussed experiment (max. WA—28.09% for the BC sample).

The highest overall acceptability was observed for the BS and PC samples. The flax seed bowls were rated high in terms of palatability and texture, i.e., the experience received during consumption. Taste preferences are considered to be the most important in the sensory evaluation [29]. It was observed that other food products fortified with flaxseed cake are considered quite attractive in terms of colour, appearance and texture [24] compared to the addition of other oilseed cakes. It should be noted, however, that for tableware in the form of plates, it will be justified to replace seeds with flaxseed cake. The PC sample was rated high in terms of colour and aroma holistically. In addition, the obtained edible tableware did not contain any added sweeteners.

## 5. Conclusions

The widespread use of disposable packaging or tableware, often not recyclable, leads to the creation of an increasing amount of waste and thus constitutes a serious ecological problem. The conducted experiment highlights the potential of edible tableware based on flax seeds or cake as a sustainable alternative to plastic. The research results indicate good mechanical properties of edible bowls and plates. The containers obtained were tight and were not damaged under force or during soaking. These tableware are generally well accepted by consumers. The next stage of the research may be the selection of appropriate additives improving the taste and smell of the biomaterial.

## Figures and Tables

**Table 1:** Model of experiment parameters.

Probe Code	PS	BS	PC	BC
**Form**	plate	bowl	plate	bowl
**Ingredients [g]**				
Flaxseed	300	300	-	-
Flaxseed cake	-	-	300	300
Rapeseed oil	54	54	54	54
Wheat flour	55	55	55	55
Salt	0.4	0.4	0.4	0.4
Pepper	0.2	0.2	0.2	0.2
Cinnamon	0.2	0.2	0.2	0.2
Carob	6.6	6.6	6.6	6.6
Flax mucilage	70	70	70	70

PS—flaxseed plates, BS—flaxseed bowl, PC—flaxseed cake plates, BC—flaxseed cake bowl.

**Table 2:** Variations in the physical properties of edible tableware.

Probe	w [%]	CL [%]	L*	a*	b*
PS	44.78 ± 2.69 ^a^	0.06 ± 0.01 ^b^	27.66 ± 1.24 ^b^	4.18 ± 0.29 ^c^	4.66 ± 0.38 ^c^
BS	13.65 ± 1.09 ^c^	0.08 ± 0.01 ^b^	28.08 ± 0.15 ^b^	4.70 ± 0.50 ^bc^	5.13 ± 0.26 ^c^
PC	34.04 ± 3.86 ^b^	0.12 ± 0.03 ^a^	30.20 ± 0.30 ^a^	5.09 ± 0.21 ^b^	6.26 ± 0.71 ^b^
BC	10.74 ± 1.31 ^c^	0.09 ± 0.01 ^ab^	29.77 ± 0.40 ^a^	5.75 ± 0.12 ^a^	7.42 ± 0.30 ^a^

w—change in the height of the tableware; CL—crumbling losses; L*,a*, b*—colour parameters: L—lightness; a—green (−), red (+); b—blue (−), yellow (+). The data value of each parameter with different superscript letters in the columns are significantly different (Tukey test. *p* ≤ 0.05).

**Table 3:** The strength of edible tableware.

Probe	PS	BS	PC	BC
Before using	4.87 ± 0.21 ^bc^	5.52 ± 0.66 ^b^	7.71 ± 0.54 ^a^	5.17 ± 0.61 ^bc^
After using	4.57 ± 0.40 ^c^	2.48 ± 0.08 ^e^	3.53 ± 0.38 ^d^	2.22 ± 0.32 ^e^

The data value of each parameter with different superscript letters in the columns are significantly different (Tukey test. *p* ≤ 0.05).

## Data Availability

Data are contained within the article.
